# Supplementary material for: Iterative improvement in the automatic modular design of robot swarms
Source: PeerJ Comput Sci. 2020 Dec 7;6:e322. doi: 10.7717/peerj-cs.322 (PMC7924708; doi:10.7717/peerj-cs.322)
Supplement: Supplemental Information 3 [file peerj-cs-06-322-s003.zip › argos3/doc/api/standalone/a00442_source.html]

ARGoS: plugins/robots/foot-bot/control\_interface/ci\_footbot\_gripper\_actuator.cpp Source File


- Main Page
- Related Pages
- Namespaces
- Classes
- Files

- File List
- File Members

# plugins/robots/foot-bot/control\_interface/ci\_footbot\_gripper\_actuator.cpp

Go to the documentation of this file.

```
00001 
00007 #include "ci_footbot_gripper_actuator.h"
00008 
00009 #ifdef ARGOS_WITH_LUA
00010 #include <argos3/core/wrappers/lua/lua_utility.h>
00011 #endif
00012 
00013 namespace argos {
00014 
00015   /****************************************/
00016   /****************************************/
00017    
00018   CRadians CCI_FootBotGripperActuator::LOCKED_NEGATIVE(-ARGOS_PI * 0.5f);
00019   CRadians CCI_FootBotGripperActuator::LOCKED_POSITIVE(ARGOS_PI * 0.5f);
00020   CRadians CCI_FootBotGripperActuator::UNLOCKED(0.0f);
00021 
00022   CRange<CRadians> APERTURE_RANGE(CCI_FootBotGripperActuator::LOCKED_NEGATIVE,
00023                                   CCI_FootBotGripperActuator::LOCKED_POSITIVE);
00024    
00025   /****************************************/
00026   /****************************************/
00027 
00028 #ifdef ARGOS_WITH_LUA
00029   /*
00030    * The stack must have no values
00031    */
00032   int LuaGripperLockPositive(lua_State* pt_lua_state) {
00033     /* Get wheel speed from stack */
00034     if(lua_gettop(pt_lua_state) != 0) {
00035       return luaL_error(pt_lua_state, "robot.gripper.lock_positive() expects no arguments");
00036     }
00037     /* Perform action */
00038     CLuaUtility::GetDeviceInstance<CCI_FootBotGripperActuator>(pt_lua_state, "gripper")->LockPositive();
00039     return 0;
00040   }
00041 
00042   /*
00043    * The stack must have no values
00044    */
00045   int LuaGripperLockNegative(lua_State* pt_lua_state) {
00046     /* Get wheel speed from stack */
00047     if(lua_gettop(pt_lua_state) != 0) {
00048       return luaL_error(pt_lua_state, "robot.gripper.lock_negative() expects no arguments");
00049     }
00050     /* Perform action */
00051     CLuaUtility::GetDeviceInstance<CCI_FootBotGripperActuator>(pt_lua_state, "gripper")->LockNegative();
00052     return 0;
00053   }
00054 
00055   /*
00056    * The stack must have no values
00057    */
00058   int LuaGripperUnlock(lua_State* pt_lua_state) {
00059     /* Get wheel speed from stack */
00060     if(lua_gettop(pt_lua_state) != 0) {
00061       return luaL_error(pt_lua_state, "robot.gripper.unlock() expects no arguments");
00062     }
00063     /* Perform action */
00064     CLuaUtility::GetDeviceInstance<CCI_FootBotGripperActuator>(pt_lua_state, "gripper")->Unlock();
00065     return 0;
00066   }
00067 #endif
00068 
00069   /****************************************/
00070   /****************************************/
00071 
00072   CCI_FootBotGripperActuator::CCI_FootBotGripperActuator() :
00073     m_cAperture(UNLOCKED) {}
00074 
00075   /****************************************/
00076   /****************************************/
00077 
00078   void CCI_FootBotGripperActuator::SetAperture(const CRadians& c_aperture) {
00079     m_cAperture = c_aperture;
00080     m_cAperture.SignedNormalize();
00081     APERTURE_RANGE.TruncValue(m_cAperture);
00082   }
00083 
00084   /****************************************/
00085   /****************************************/
00086 
00087   void CCI_FootBotGripperActuator::LockPositive() {
00088     SetAperture(LOCKED_POSITIVE);
00089   }
00090 
00091   /****************************************/
00092   /****************************************/
00093 
00094   void CCI_FootBotGripperActuator::LockNegative() {
00095     SetAperture(LOCKED_NEGATIVE);
00096   }
00097 
00098   /****************************************/
00099   /****************************************/
00100 
00101   void CCI_FootBotGripperActuator::Unlock() {
00102     SetAperture(UNLOCKED);
00103   }
00104 
00105   /****************************************/
00106   /****************************************/
00107 
00108 #ifdef ARGOS_WITH_LUA
00109   void CCI_FootBotGripperActuator::CreateLuaState(lua_State* pt_lua_state) {
00110     CLuaUtility::OpenRobotStateTable (pt_lua_state, "gripper"                               );
00111     CLuaUtility::AddToTable          (pt_lua_state, "_instance",     this                   );
00112     CLuaUtility::AddToTable          (pt_lua_state, "lock_positive", &LuaGripperLockPositive);
00113     CLuaUtility::AddToTable          (pt_lua_state, "lock_negative", &LuaGripperLockNegative);
00114     CLuaUtility::AddToTable          (pt_lua_state, "unlock",        &LuaGripperUnlock      );
00115     CLuaUtility::CloseRobotStateTable(pt_lua_state                                          );
00116   }
00117 #endif
00118 
00119   /****************************************/
00120   /****************************************/
00121 
00122 }
```

---

Generated on 10 Jul 2018 for ARGoS by 
 1.6.1
